# Cement-Induced Chemical Burn in a Middle-Aged Male: A Case Report and Review of Literature

**DOI:** 10.7759/cureus.53636

**Published:** 2024-02-05

**Authors:** Hafsa Zahoor, Nismat Javed, Jacob K Epperson, Darshana Ganguly, Susan Chung, Misbahuddin Khaja

**Affiliations:** 1 Internal Medicine, BronxCare Health System, Icahn School of Medicine at Mount Sinai, Bronx, USA; 2 Internal Medicine, American University of the Caribbean School of Medicine, Cupecoy, SXM; 3 Surgery, BronxCare Health System, Icahn School of Medicine at Mount Sinai, Bronx, USA; 4 Division of Pulmonary Medicine and Critical Care, Department of Internal Medicine, BronxCare Health System, Icahn School of Medicine at Mount Sinai, Bronx, USA

**Keywords:** chemical injury, burn, cement, outcomes, management

## Abstract

This case study reviews a 48-year-old Hispanic male working in construction who presented with left upper medial thigh pain, redness, and swelling after exposure to hazardous chemicals during cement processing. Initially diagnosed with cellulitis and adjacent myositis, the patient met sepsis criteria and received empiric antibiotics. However, negative cultures and an evolving wound appearance shifted the diagnosis towards bullous diseases and chemical injury. Occupational history and physical exam findings pointed towards injury secondary to chemical exposure, common in cement workers with inadequate protective gear. Cement burns, often insidious, are underreported due to their slow progression, mainly affecting the lower extremities. These burns involve chemical, mechanical, and hypersensitivity mechanisms that can mimic infection on imaging. This case highlights the importance of recognizing and managing cement burns promptly, emphasizing protective measures, decontamination, and potential early intervention by burn specialists.

## Introduction

Chemical burns from cement are widely acknowledged and prevalent, especially in industrialized countries. These burns can be categorized as heat, abrasion, or explosive burns [1]. Abrasions mostly occur due to prolonged skin contact and rubbing with alkaline cement [1,2]. It is the most common type of chemical burn associated with cement exposure [1,2]. Heat burns result from contact with hot cement powder during the manufacturing process [1,2]. This suggests that individuals involved in the production of cement are at risk of sustaining heat burns. Explosive burns are caused by the sudden discharge of powder from a kiln during the cement manufacturing process [3]. It implies that there is a risk of explosive events during cement production that can lead to burns. The incidence and prevalence estimates of the disorder are relatively limited. Management of the burn is mostly supportive with fluid resuscitation and treatment for sepsis. Early removal and grafting of extensive and deep burns is strongly advocated. For smaller areas of deep wounds, meshed autografts are often used, while larger deep wounds are addressed through a technique involving the intermingled transplantation of autograft and homograft pieces [2]. When deep wounds impact functional areas such as the hands and face, the approach involves covering them with sheets of autograft as much as possible to enhance both functional recovery and cosmetic appearance [2]. In cases where there is extensive muscle necrosis, there may be a necessity to amputate certain parts of a limb in order to preserve the patient's life. Considering the complications associated with the disease and its prolonged course, it is important to highlight this pathology. The case report presented involves a 48-year-old male who initially presented with left-sided thigh pain and eventually developed a chemical burn. This case serves as an illustration of the potential consequences of cement exposure and highlights the need for awareness and proper management of such incidents.

## Case presentation

**Figure 1:**
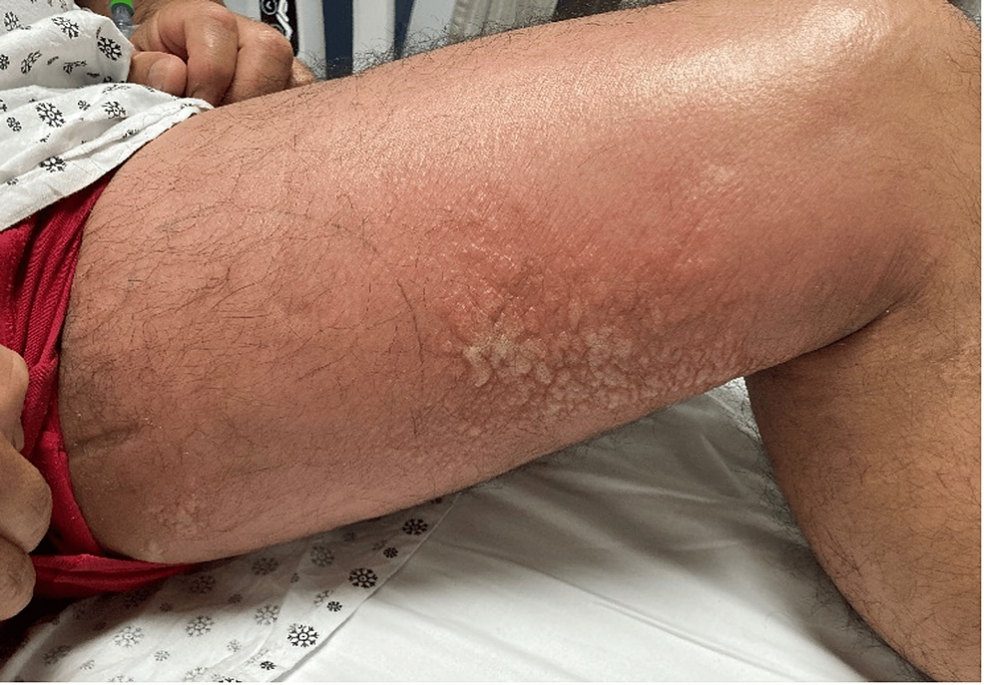
Blisters observed in the upper thigh

**Figure 2:**
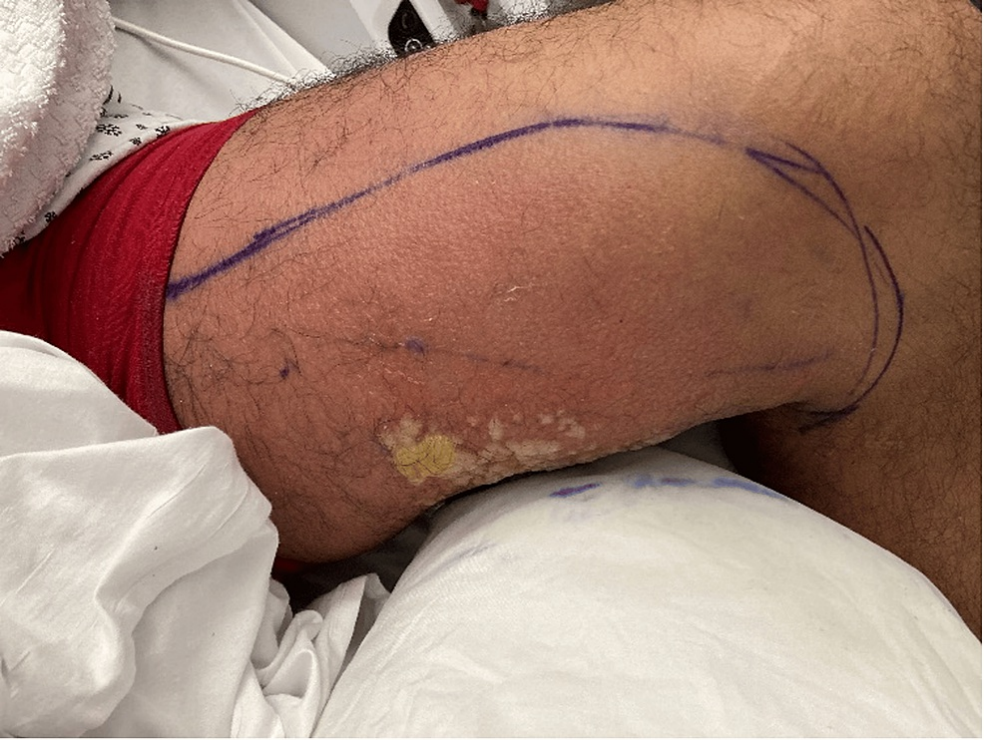
Pus filling blisters with marking around erythema

This is a 48-year-old Hispanic male who worked in construction and presented to the emergency department with no known past medical history and a complaint of left upper medial thigh pain for the past two days. The pain was gradual in onset. It was non-radiating, non-shifting throbbing pain with associated redness and swelling of the affected area. He mentioned that he was exposed to hazardous chemicals while processing cement and wearing insufficient protective gear. He denied any trauma to the site, insect bites, fever, chills, rigor, or changes in bladder or bowel habits. In the emergency department, his vital signs were unremarkable. A physical exam showed left upper medial thigh tenderness to palpation, but there was no erythema, fluctuance, or crepitus noted (Figure 1,2).

Lab investigations revealed leukocytosis with an elevated C-reactive protein. A CT of lower extremities with intravenous contrast revealed subcutaneous edema in the medial thigh with reactive appearing nodes in the lower inguinal region and upper medial thigh. The patient was admitted for further management. He was started on vncomycin and piperacillin-tazobactam (Zosyn). Repeat CT of the extremity showed severe cellulitis and fasciitis of the thigh, severe myositis of the extensor muscles of the knee, severe cellulitis of the visualized portions of the leg, and left external iliac lymphadenopathy in the pelvis. No abscess was noted. Clindamycin was added to the regimen.  

Dermatology and Infectious Disease were consulted. The patient developed bullae over the region of the previous cellulitis, with the expulsion of clear fluid. The base was noted to appear yellow and covered in pus. Coverage for Herpes Zoster with Valacyclovir and topical mupirocin was started, and the patient was placed in airborne isolation. 

Physical examination by Plastic Surgery at the time showed an edematous and erythematous left medial thigh suggestive of a caustic injury or liquefactive necrosis. Saponified blisters appearing in a small band surrounded by a red rash and induration without fluctuance strongly suggested a chemical burn. The antibiotic regimen was modified; Zosyn was discontinued and meropenem was added for empirical coverage.  There was low suspicion of viral or fungal infection. Over the next 5 days, the blisters and erythema continued to improve, and the blisters resolved by day 10. The patient was discharged on day 11 with a 7-day course of clindamycin, Augmentin, topical mupirocin, and instructions to follow up with Plastic Surgery.  

## Discussion

In our case, the patient fulfilled SIRS criteria and was empirically treated for cellulitis with adjacent myositis. Although blood cultures were negative, empiric antibiotic coverage for methicillin-susceptible *Staphylococcus aureus* (MSSA) and Group A beta-hemolytic Streptococci were started, as they account for more than 70% of patients with cellulitis [4]. In this instance, vancomycin, piperacillin-tobramycin, and clindamycin were selected at various times for empiric coverage. With consistently negative blood and wound cultures and the evolution of the wound appearance, the differential diagnosis shifted towards bullous diseases and chemical injury. Bullous diseases can be divided into two classifications, namely localized, or generalized. Localized diseases include herpes zoster, herpes simplex virus, contact dermatitis, and mild dermatitis herpetiformis. Generalized diseases include diseases such as pemphigus vulgaris, toxic epidermal necrolysis, bullous pemphigoid, or Sweet syndrome [5]. As the affected area was limited to the medial thigh, generalized bullous diseases were excluded from the differential diagnosis. Although the suspicion of herpes zoster was low, empiric coverage with antivirals was started. 

When factoring in the patient's occupational history and physical exam findings, contact dermatitis and chemical injury appeared most likely. Chemical injury has also been documented previously with relatively benign chemicals; for example, the ink used in voting in a study based in India resulted in partial thickness burns [6]. In our study, chemical injury complicated by liquefactive necrosis was the probable clinical diagnosis. With suboptimal personal protective gear and exposure to construction materials on site, chemical burns seemed very likely. Wet cement injuries are an often under-reported type of injury in construction workers. Cement, which has a pH ranging from 10 to 14, can form slowly progressing alkali burns which often go unnoticed for up to 48 hours (about 2 days), or until the full-thickness burn has become evident [7, 8].  

The insidious nature of cement burns, as previously stated, is often underreported. A summary of these cases is shown in Table 1 [9-21]. 

**Table 1:** Summary of the cases M-male, F-female, THR-Total Hip Replacement, TKA-Total Knee Arthroplasty

Author	Age/Gender	Features	Management	Outcome
Morley et al [9], 1996.	43/M, 44/M	sustained partial-thickness burns to arms and upper thighs. The total surface burned was estimated at 41% (x1) full-thickness burns right hand, neck and right calf and partial thickness burns to his left knee. The burn was estimated at 8% (x1)	Fluids and analgesia (x2) Split skin grafting (x1) and full thickness grafting (x1)	Alive (x2)
Burston et al [10]., 2007	63/F after THR with cement cast	Two deep dermal defects measured 2 cm × 3 cm.	Fluids and analgesia Hydrocolloid dressings	Alive
Abola et al [11] ., 2013	61/F after TKA	3 cm by 3 cm defect that was a third degree burn on popliteal fossa	Grafting	Alive
Chiriac et al [12]., 2016	54/M construction worker	Edema and necrotic crusting noted	Escharotomy of the lower limbs was performed, systemic antibiotics	Alive
Seyhan et al [13] ., 2012	53/F	Second-degree burn on bilateral ankles	Split-thickness skin graft	Alive
Waage et al [14]., 2014	50/M	Second- and third-degree burns to both his lower legs about 5% of the body surface	Partial skin thickness graft	Alive
Barr et al [15]., 2002	26/M	4 cm dorsal laceration exposing the index metacarpal and extending radially into the first web space with extensive necrosis	Full-thickness skin graft	Alive with residual weakness in the region
Skiendzielewski et al [16]., 1980	24/M	First- and second-degree burns of both lower extremities	Debridement of debris	Alive
Lim et al [17]., 2006	20/M	Conjunctival epithelial defect with denuded stromal with an elevated edge	Repetitive debridement and topical eye drops	Alive with mild impairment of visual acuity
Arumilli et al [18]., 2007	31/M after excision of bone tumor and placement of bone cement for healing	Well-defined eschar measuring 6 × 4 cm over the knee	Debridement	Alive
Biello et al [19]., 2021	40/F, 68/F	Fragments of calcified bone cement and granulation tissue in the sphenoid sinus and inflammation of the mucosal lining (x2)	Excision and debridement	Alive (x2)
Peters et al [20]., 1984	59/M	Second- and third-degree burns over the ankle	Skin grafting	Alive
Mehta et al [21]., 2002	47/M	Third-degree burn over the ankle, 10% of the total area	Skin grafting	Alive

This is partly due to the slow evolution of the injury. Most cement-related injuries are localized to the lower extremities, the feet and ankles being the most common sites, with a total body surface area of rarely greater than 5%. The typical presentation of the injury involves a slowly penetrating yet painless necrosis of the skin tissue, with the only noticeable symptom often being redness and warmth of the affected area. The pain associated with such burns is often attributed to fluid build-up from the injured tissue [22].  

Cement causes injuries in a multifactored mechanistic fashion, the first being a chemical alkalotic injury, the second a mechanical abrasion, and the last being a type IV hypersensitivity reaction. Most dry cement powders contain chemicals such as calcium oxide, which is very hygroscopic [23, 24]. As a powder, it sticks to sweat-moistened skin and the small grain size causes microabrasions which expose the sensitive underlying tissue. When moisture from sweat and skin is added, it becomes calcium hydroxide through an exothermic reaction, which raises the pH, thereby becoming caustic to the skin epithelium [25, 26]. A type IV hypersensitivity reaction associated with dermatitis is caused by hexavalent chromate ions and irritation from the sand aggregate [26]. This intracellular edema and disruption of intercellular adhesion causes exocytosis of leukocytes and eosinophils into spongiotic foci. This can often be confused with infection on various forms of imaging. When spongiosis becomes more severe, acantholysis can occur, forming a bulla [23]. 

Cement burns are often underestimated in terms of their prevalence, whether it be due to victims' lack of awareness of exposure, the healthcare providers' failure to recognize the injury, or similar features to other differentials, like infection. These burns are essentially chemical injuries in nature, which emphasizes the importance of personal protective equipment and immediate decontamination if exposed. 

Management strategies involve controlling the source, such as removal of cement, to prevent ongoing injury. Decontamination involves thorough irrigation with normal saline, although alternatives like polyethylene glycol, isopropyl alcohol, or dextrose can be used, though their superiority in clinical outcomes remains unproven. While the literature about high- versus low-pressure irrigation for cement burns is limited, the use of high-pressure irrigation may worsen tissue damage leading to further complications. Although copious water lavage is the first line for virtually all chemical burns, the calcium oxide in cement powders can react to the water and create significant exothermy, which may worsen injury [24]. Dry cement mixtures should be brushed off first. If the pH is high enough, neutralizing with a weak acid, such as muriatic can be warranted [26]. Antibiotics are generally not indicated but should be used if there is concern for underlying cellulitis. Patients should ultimately be assessed by a burn specialist for potential early debridement and split-thickness skin grafting, if necessary. 

## Conclusions

Chemical burns secondary to cement pose a variety of problems as well as complications including limited assessment of the burn width and limitations in management using conservative agents. Chemical injury induced by cement is one of the many severe reactions that can occur as a result of the exposure. It is important to determine the nature and duration of exposure for each pattern of injury; for example, chemical injury, as observed in our case, was associated with acute exposure to increased amounts of cement given the lack of protection. Therefore, there is a need to recognize and manage the pathology promptly to prevent complications. More studies are needed to determine the type of agents that are useful in management and to determine pathogenesis. Furthermore, occupational studies need to be conducted to determine the hazardous levels of cement and limit the exposure to prevent long-lasting damage. 
